# Real‐World Evaluation of Sequential Monopolar‐Bipolar RF Device for Rejuvenation

**DOI:** 10.1111/jocd.70687

**Published:** 2026-02-09

**Authors:** Nicole Veltri, Michele Frank, Morgan Richter, Saulis Banionis, Julia Bifulco, Narendra Kumar

**Affiliations:** ^1^ Beyond Beauty Medical Spa Rocky Hill Connecticut USA; ^2^ Ooh La La Spa, Antiaging, and Wellness Glen Carbon Illinois USA; ^3^ Palm Beach Anti‐Aging and Regenerative Medicine Wellington Florida USA; ^4^ Bellagena Med Spa Bradenton Florida USA; ^5^ Global Medical Affairs Jeisys Med Inc Seoul South Korea

**Keywords:** DENSITY RF, non‐invasive aesthetic procedures, radiofrequency, real‐world evidence, sequential monopolar‐bipolar

## Abstract

**Background:**

Sequential monopolar‐bipolar radiofrequency (RF) systems aim to provide multi‐layer dermal heating for skin tightening and rejuvenation. Real‐world outcome data for these devices remain limited.

**Objectives:**

To evaluate the clinical efficacy, safety, and patient‐reported outcomes of the DENSITY NOIR sequential RF device for aesthetic facial indications.

**Methods:**

A retrospective multicentre case series was conducted across dermatology clinics in the United States. Consecutive patients who underwent DENSITY NOIR RF treatment and had standardized pre‐and post‐treatment photographs, along with follow‐up surveys completed ≥ 30 days after treatment, were included in the study. Provider‐rated improvement (4‐point scale) and patient‐reported outcomes, including visible improvement, satisfaction, pain, downtime, and likelihood to recommend, were summarized descriptively. Age‐stratified subgroup analyses and correlation assessments were also performed.

**Results:**

Twenty‐five patients (88% female; age range 26–55 years) met the inclusion criteria. All demonstrated improvement from baseline; 56% showed moderate improvement, while 24% showed marked improvement. Patient satisfaction was high (100%), with 48% “very satisfied.” Likelihood‐to‐recommend scores were strong (median 10/10). Downtime was minimal; 92% reported none. Age‐subgroup analyses showed greater proportions of marked improvement and “very satisfied” responses among patients > 55 years. Pain scores were low, and no adverse events were recorded.

**Conclusions:**

Sequential RF treatment demonstrated meaningful aesthetic improvement with excellent tolerability, high satisfaction, and negligible downtime. Findings support its value as a minimally invasive skin‐rejuvenation option.

## 1 Introduction

Aging of the skin leads to collagen loss, elastin degradation, and dermal atrophy, manifesting as wrinkles, laxity, and texture changes. To address these concerns without surgery, non‐invasive energy‐based treatments have become increasingly popular for skin rejuvenation, offering improvement with minimal downtime [1, 2]. Among available modalities, radiofrequency (RF) has gained prominence due to its ability to heat dermal tissues and induce collagen remodeling with minimal epidermal injury. The thermal effect causes immediate collagen fiber contraction and initiates a wound‐healing cascade that triggers neocollagenesis over ensuing months [3].

Conventional RF devices deliver energy through either monopolar or bipolar electrodes. Monopolar RF penetrates deeply, heating the deep dermis and subcutaneous tissue for significant tightening at depth. Bipolar RF confines current to the superficial dermis, effectively improving fine rhytids and skin texture [1]. However, a single modality often cannot simultaneously tighten deep structures and refine the superficial skin.^4^ To overcome this, newer systems integrate sequential monopolar pulse that preheats deeper tissues, reducing impedance and priming the dermis, thereby enhancing the efficacy of the subsequent bipolar pulse targeted at superficial layers. This dual‐energy approach is intended to achieve synergistic, multi‐level skin tightening.

Early evidence from preclinical studies on sequential monopolar‐bipolar delivery indicates that this approach can significantly increase dermal collagen and elastin production compared to monopolar RF alone [4]. Small‐scale clinical reports have likewise noted significant skin tightening and contour improvement with this technology, with minimal pain or downtime [5]. Nevertheless, real‐world clinical outcomes and patient satisfaction data for this treatment modality are limited.

This study aimed to retrospectively evaluate the efficacy, safety, and patient‐reported outcomes of the DENSITY sequential RF treatment across various dermatologic aesthetic indications. The study objective was to describe the proportion of patients demonstrating improvements on the site's standardized 4‐point provider‐rated scale at 30–90 days post treatment.

## 2 Methods

### 2.1 Study Design and Participants

This retrospective multicentre case series evaluated real‐world outcomes of patients treated with the DENSITY NOIR sequential RF device across dermatology clinics in the United States between July and September 2025. Consecutive patients were eligible if they had undergone DENSITY NOIR RF treatment during the study period, had availability of standardized pre‐and post‐treatment photographs with ≥ 30 days of follow‐up, and completed a structured post‐treatment survey. Patients were excluded if they received concurrent aesthetic procedures during the interval or if their photographic or survey data were incomplete.

### 2.2 Interventions

All participants received a single DENSITY NOIR RF treatment administered using a standardized clinical protocol. After gentle cleansing and application of coupling gel, the hand piece was applied to the designated treatment area (primarily the face). Sequential monopolar and bipolar RF pulses were delivered in multiple passes to achieve moderate warmth and mild erythema as the desired clinical endpoint. No typical or systemic anesthesia was required, reflecting the generally comfortable nature of the procedure. Post‐procedure care consisted of routine moisturization and photoprotection; no downtime‐specific instructions were necessary.

### 2.3 Treatment Protocol

The DENSITY NOIR (Jeisys Medical Inc., Seoul, South Korea) delivers monopolar and bipolar RF energy sequentially in each pulse. Treatment parameters, including RF tip selection (High F, High E, or High B), energy levels (typically 2.0–4.0), number of passes, and anatomical site, were adapted based on clinical judgment and patient tolerance. Treatment sites included the full face, lower face, submentum, periorbital region, and eyelids. Average treatment duration ranged from 30 to 60 min.

### 2.4 Data Collection

Data were extracted from routine clinical documentation, provider case report forms, and structured patient follow‐up surveys. Variables captured included demographic data, Fitzpatrick skin type, treatment area, RF tip used, energy settings, treatment duration, and consent status for photography and data use. Patient‐reported outcomes included pain (0–4 scale), visible improvement (slight/moderate/marked), overall satisfaction (“satisfied” or “very satisfied”), downtime (< 24 h or none), willingness to undergo treatment again, and likelihood of recommending the treatment (0–10 scale).

### 2.5 Outcomes and Evaluations

The primary outcome was provider‐rated global aesthetic improvement approximately 1–3 months post‐treatment, assessed using a validated 4‐point scale (none, slight, moderate, or marked improvement). Secondary outcomes comprised patient‐reported satisfaction, visible improvement, sensation during treatment, willingness to repeat the procedure, and likelihood to recommend the treatment to others. Adverse events within 30 days were identified from clinical records.

### 2.6 Statistical Analysis Plan

All analyses were performed using IBM SPSS Statistics version 27.0 (IBM Corp., Armonk, NY). Descriptive statistics summarized demographic variables and treatment outcomes. Categorical variables were reported as frequencies and percentages. Comparisons between age groups (≤ 55 vs. > 55 years) were conducted using Chi‐square or Fisher's exact tests, as appropriate. Statistical significance was set at *p* < 0.05. Correlation analyses were performed to assess associations among key outcome measures, including provider‐rated improvement, visible improvement, satisfaction, and likelihood of recommendation. All tests were two‐tailed, and results were interpreted in the context of clinical relevance.

### 2.7 Ethics and Privacy

All patient data were fully de‐identified before analysis. Participating sites confirmed that patients had provided consent for the use of their images and anonymized data. Given the retrospective design and use of standard‐of‐care procedures, this study met criteria for minimal‐risk research and qualified for a waiver of informed consent.

## 3 Results

### 3.1 Participant Characteristics

A total of 25 patients (22 women, 3 men) met the inclusion criteria. The majority had Fitzpatrick skin types II‐III and were treated for facial aesthetic concerns, including laxity, wrinkles, and acne scarring. Treatment duration ranged from 30 to 60 min for most patients. The distribution of Fitzpatrick skin types and RF tips used is shown in Figures 1a and 1b. Demographic details are summarized in Table 1. Follow‐up evaluations occurred at a median of 60 days post‐treatment (range: 30–90 days).

**FIGURE 1a:**
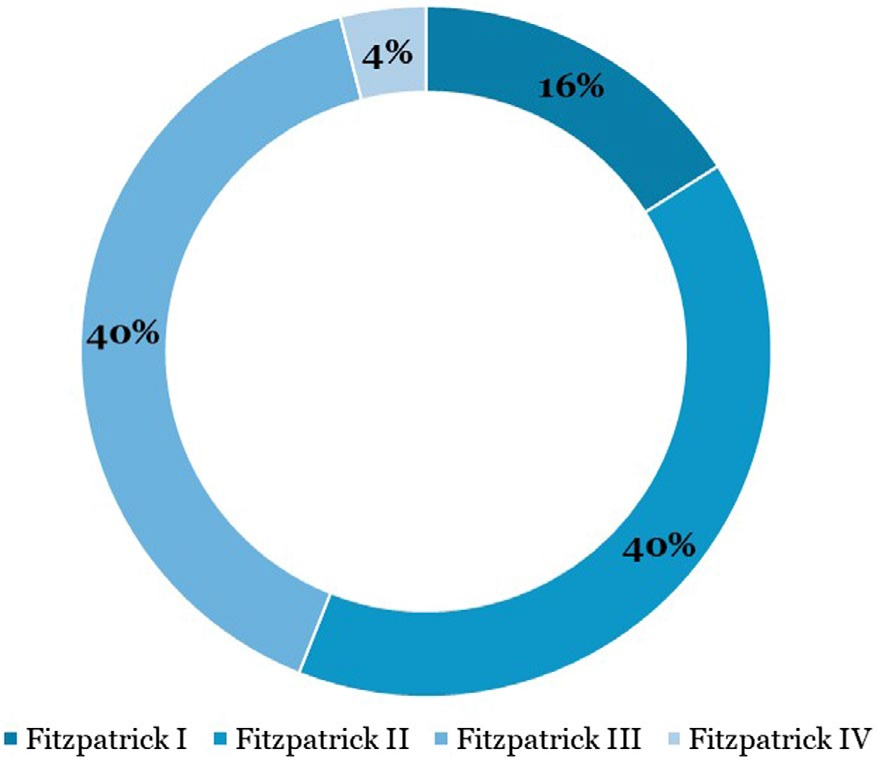
Fitzpatrick skin type distribution.

**FIGURE 1b:**
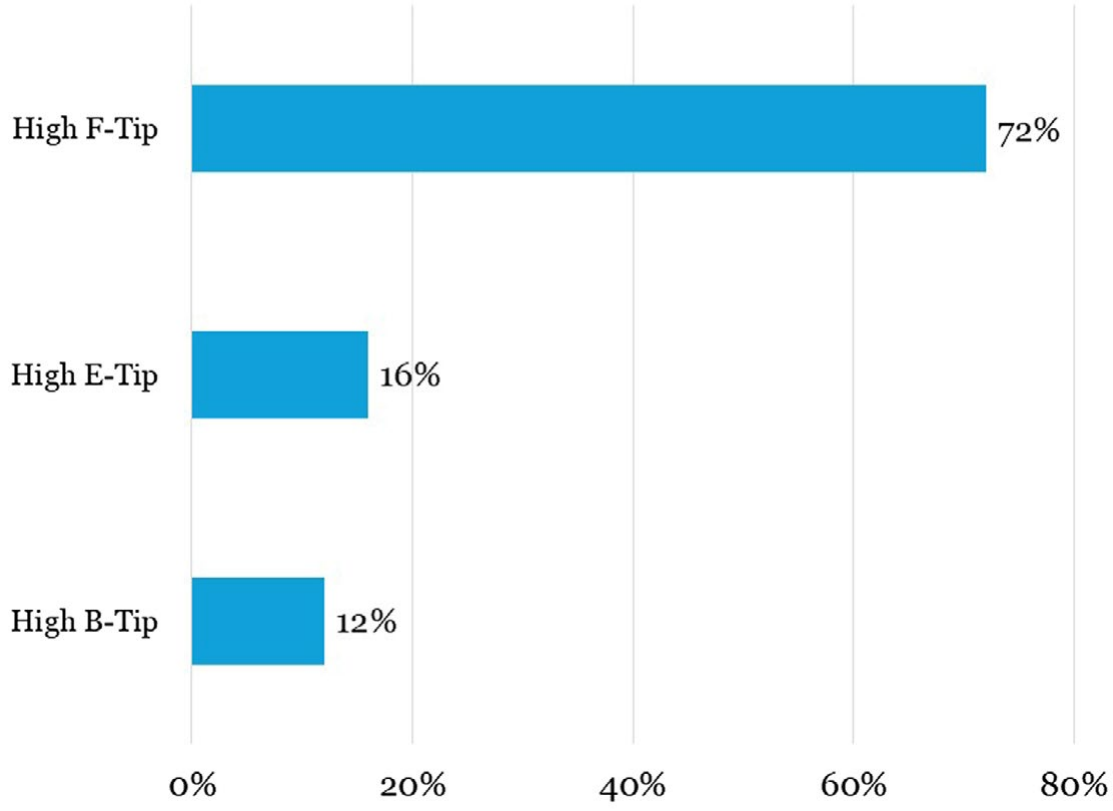
DENSITY treatment tip type distribution.

**TABLE 1:** Demographic details of study participants.

Patient characteristics	*n*
Fitzpatrick skin type	
I	4
II	10
II	10
IV	1
Age	
> 55	18
46–55	4
36–45	2
26–35	1
Gender	
Male	3
Female	22
Treatment duration	
0–30 min	4
30–60 min	18
60–90 min	3
DENSITY tip used to perform treatment	
High F Tip	18
High E Tip	4
High B Tip	3

### 3.2 Provider‐Rated Aesthetic Improvement

**FIGURE 2:**
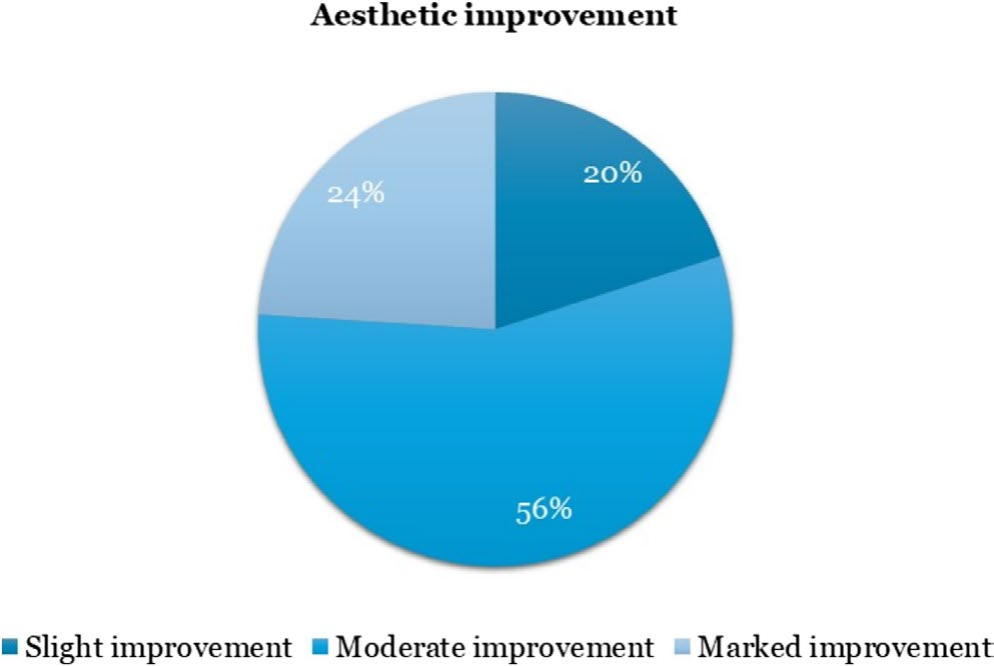
Participant's assessment of aesthetic improvement after treatment.

All patients demonstrated improvement compared with baseline. Providers rated 56% of patients as having moderate improvement and 24% as having marked improvement, while the remaining 20% showed slight improvement (Figure 2).

No patient was rated as having no improvement. Representative clinical photographs from two patients are shown to contextualize the quantitative findings. Before‐and‐after images are presented in Figures 3a and 3b. These images demonstrate the characteristic improvements seen with sequential monopolar‐bipolar RF, including enhanced lower‐face contour, softening of perioral and mid‐face lines, and improvement in facial harmony. Both cases reflect the magnitude and pattern of change commonly observed across the cohort.

**FIGURE 3a:**
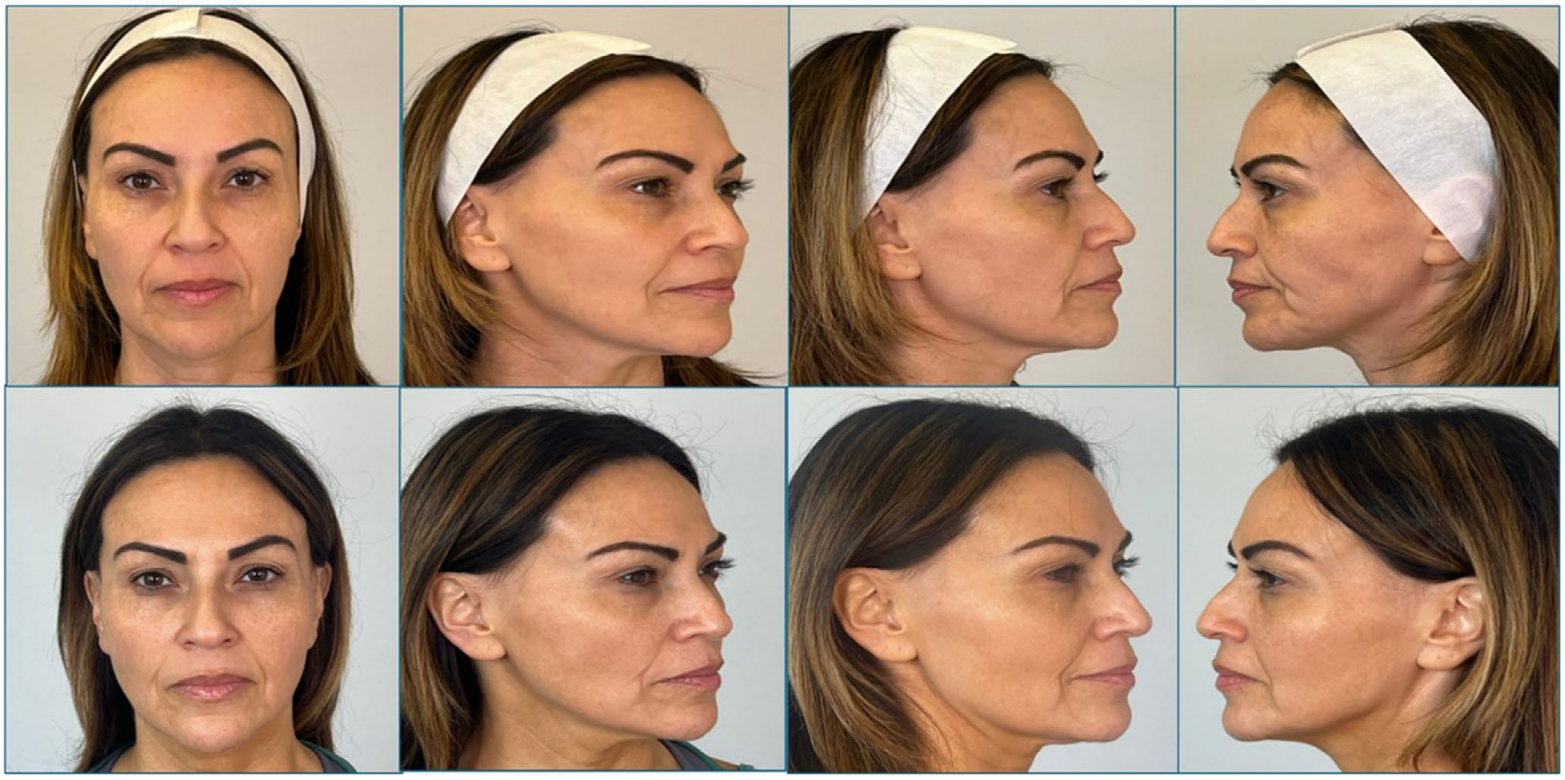
Before and 60‐day post‐treatment photographs of patient 1 following a single DENSITY sequential monopolar‐bipolar RF session, demonstrating visible improvements in jawline definition, lower‐face contouring, and perioral skin texture.

**FIGURE 3b:**
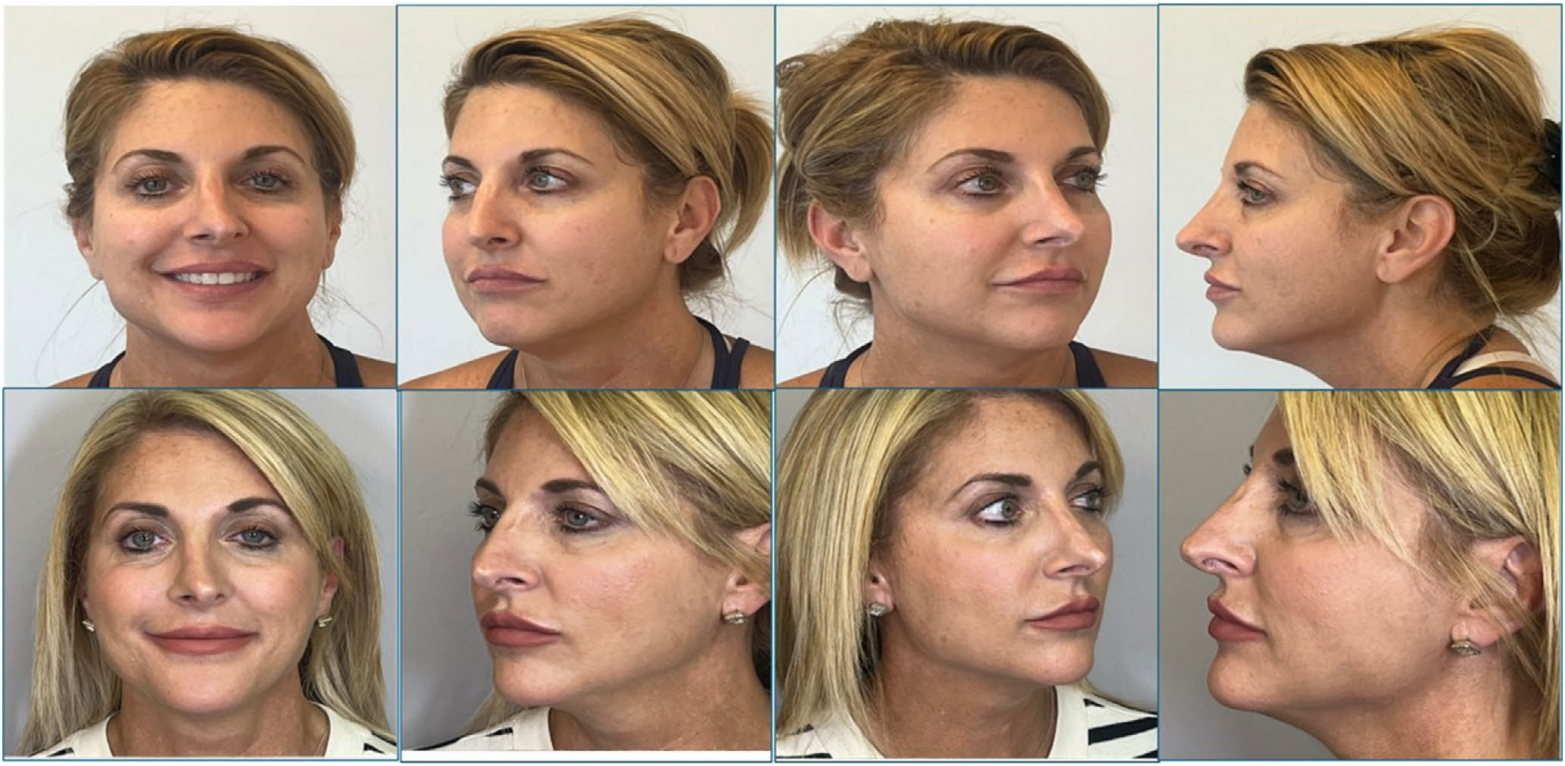
Before and 60‐day post‐treatment photographs of patient 2 showing improvements in mid‐face volume appearance, nasolabial fold softening, perioral texture, and mandibular contour.

### 3.3 Patient‐Perceived Visible Improvement

**FIGURE 4:**
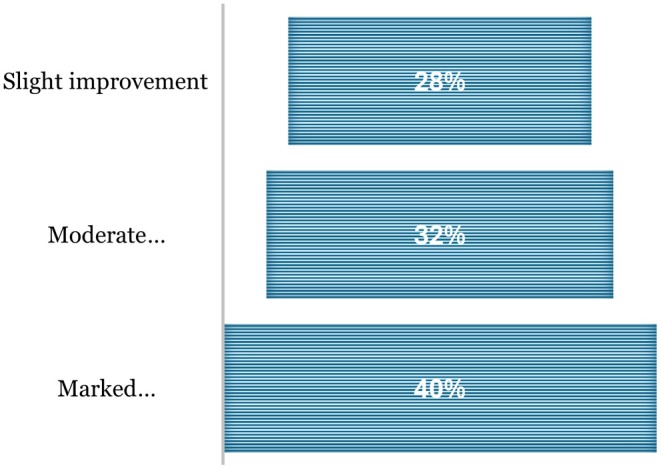
Visible improvement outcome post‐treatment.

Patient self‐assessments mirrored provider evaluations. At follow‐up, 40% of patients reported marked visible improvement, and 32% reported moderate improvement, indicating progressive enhancement in facial appearance within 1–3 months (Figure 4).

### 3.4 Patient Satisfaction and Willingness

Satisfaction levels were uniformly high. All participants were satisfied with their results, including 48% who were “very satisfied.” 100% indicated willingness to undergo the treatment again (76% “definitely yes,” 24% “probably yes”). Recommendation intent was equally strong; 64% rated their likelihood to recommend the treatment as 10/10, and the median rating was 10 (Table 2).

**TABLE 2:** Patient‐reported satisfaction and willingness outcomes.

Patient experience	Percent (%)	Frequency (*n*)
Overall satisfaction		
Satisfied	52%	13
Very satisfied	48%	12
Undergo a DENSITY treatment again		
Definitely Yes	76%	
Probably Yes	24%	
Recommend this treatment to a friend		
Definitely Yes	76%	19
Probably Yes	24%	6
How likely are you to recommend DENSITY to a friend or colleague?*		
6	4%	1
7	16%	4
8	8%	2
9	8%	2
10	64%	16

* On a scale of 0–10, where 0 is not at all likely, and 10 is extremely likely.

### 3.5 Age‐Based Subgroup Analysis

Comparisons between age groups showed meaningful trends; marked provider‐rated improvement was reported to be 50% in > 55 years vs. 14.3% in ≤ 55 years. “Very satisfied” responses were 61.1% in > 55 years vs. 14.3% in ≤ 55 years (*p* = 0.035). Sensation scores of 3–4 (moderate‐marked warmth) were reported only in the older group, though differences were not statistically significant. Likelihood‐to‐recommend scores remained high across both groups.

### 3.6 Correlation Between Improvement, Satisfaction, and Recommendation Likelihood

**FIGURE 5:**
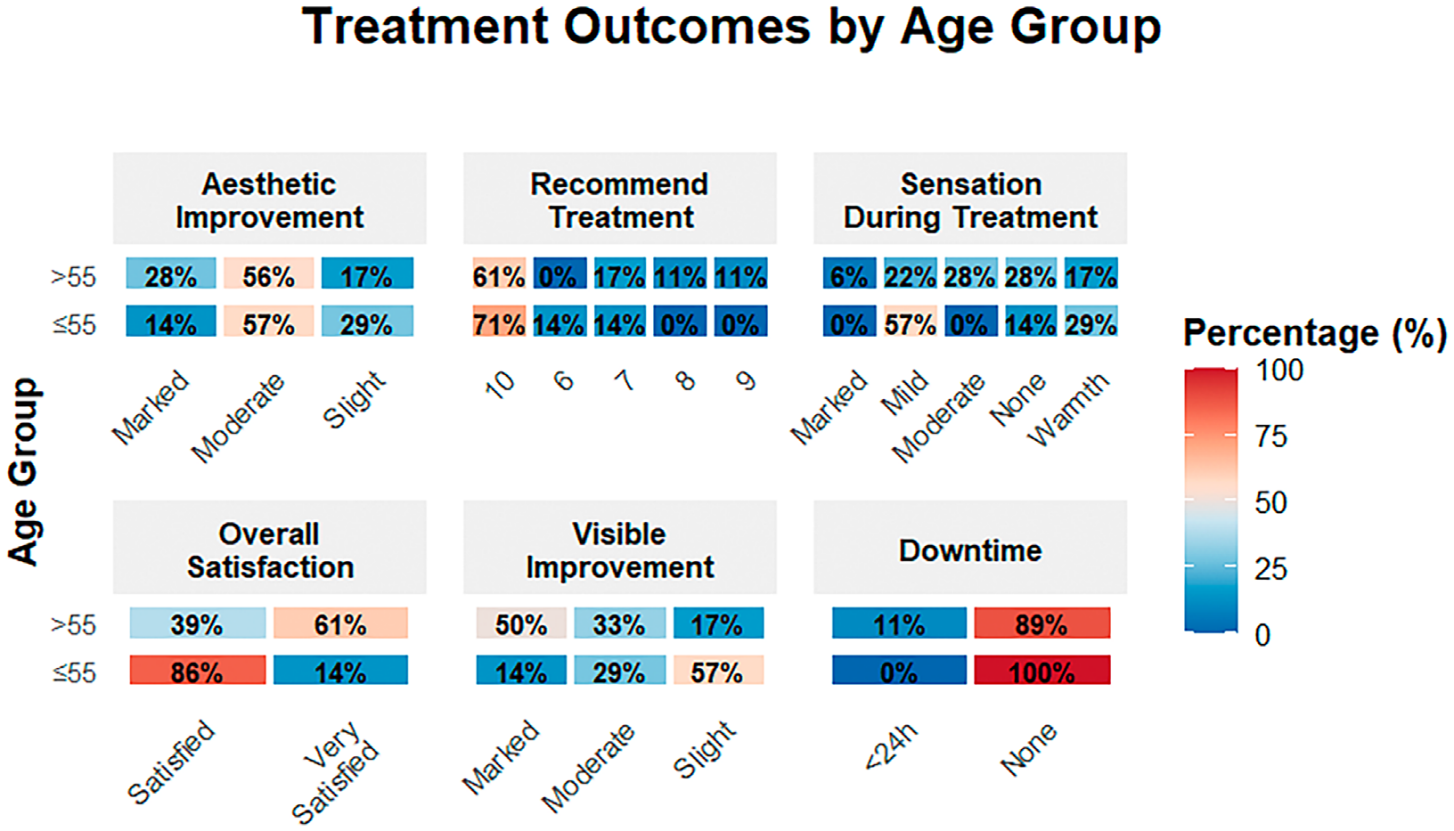
Heat‐map visualization of patient‐reported treatment outcomes stratified by age group (≤ vs. > 55 years). The figure summarizes distributions across six domains: aesthetic improvement, likelihood to recommend treatment, sensation during treatment, overall satisfaction, visible improvement, and post‐procedure downtime. Darker shades represent higher percentages within each response category. Overall, both age groups reported favorable outcomes, with notable differences in recommendation rates, perceived improvement, and downtime.

Correlation analysis revealed strong positive associations between patient‐perceived visible improvement, overall satisfaction, and the likelihood of recommending the treatment. Provider‐rated improvement also demonstrated a moderate positive correlation with the likelihood of recommendation. Pain scores showed a weak negative correlation with satisfaction, consistent with the procedure's high tolerability (Figure 5).

### 3.7 Treatment Tolerability and Safety

**FIGURE 6:**
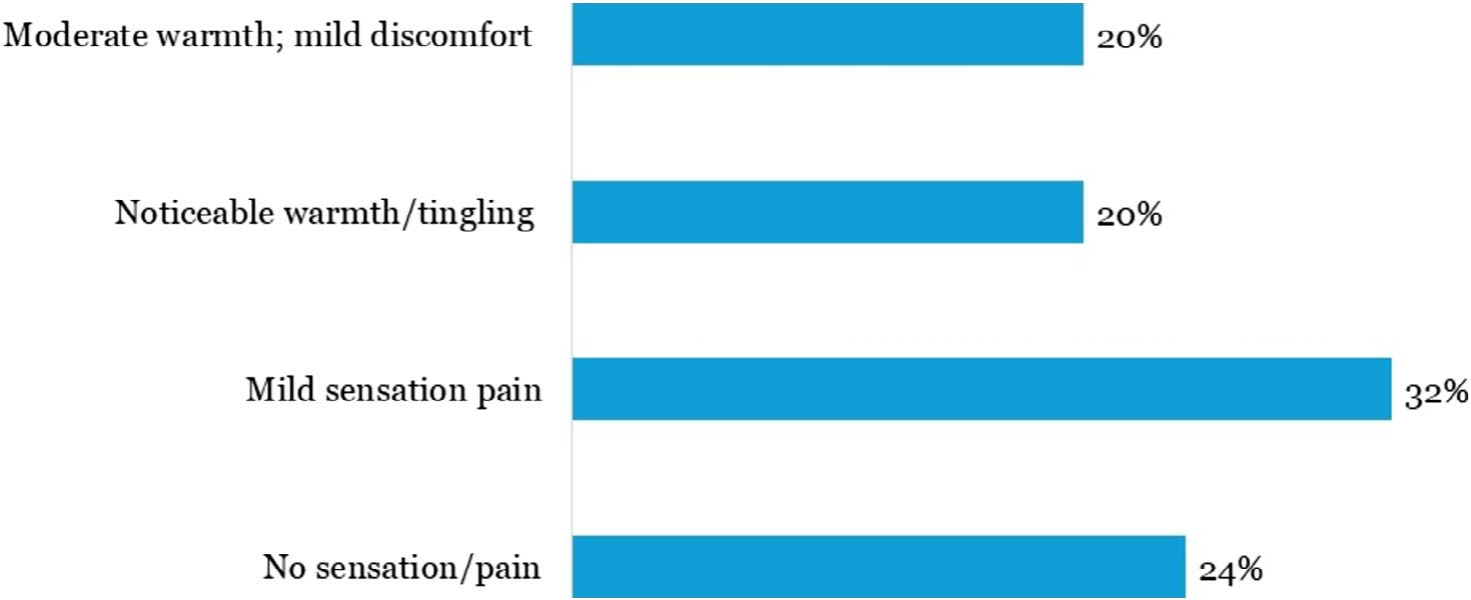
Patient‐reported pain or sensation during treatment (0–4 scale).

The treatment was well tolerated. Most patients (76%) reported no pain or only mild sensations (scores 0–2). Only one patient (4%) described it as uncomfortable (score 4). Downtime was minimal; 92% experienced no downtime, and the remaining patients reported < 24 h of mild erythema or swelling. No adverse events, including burns, scarring, or pigmentary changes, were observed during the 30‐day follow‐up (Figure 6).

## 4 Discussion

This real‐world multicentre series shows that sequential monopolar‐bipolar RF treatment with the DENSITY NOIR results in meaningful aesthetic improvements. The procedure also demonstrates a favorable safety and tolerability profile. By 1–3 months after a single treatment, all patients showed at least slight improvement, and most achieved moderate or marked improvements in facial skin appearance. These findings are consistent with previous studies demonstrating the efficacy of RF for skin tightening and rejuvenation [3]. The bipolar component may have contributed to the higher rate of marked improvement by targeting superficial dermal laxity and fine wrinkles that monopolar‐only systems may not fully address [4]. Our findings parallel those of a recent clinical case series by Oku et al., in which 15 of 16 patients treated with a similar sequential RF protocol demonstrated global improvement. Notably, no significant adverse events were reported [6]. Taken together, the evidence suggests that the dual‐energy RF reliably achieves at least moderate cosmetic improvement in appropriate patients.

Patient satisfaction was uniformly high, underscoring the importance of comfort and aligned expectations in aesthetic procedures. Patient satisfaction following non‐ablative radiofrequency treatments has varied across the literature. Individual reports have noted satisfaction rates of 82%, comprising 58% extremely satisfied/satisfied and 24% slightly satisfied [7]. In contrast, bipolar RF studies have demonstrated consistently higher satisfaction, with up to 96% of treated patients (*n* = 192) reporting satisfaction across large clinical cohorts [8]. More recent large clinical series and prospective studies evaluating contemporary monopolar and bipolar RF systems similarly report high patient satisfaction and favorable tolerability profiles [9]. In this context, the uniformly high satisfaction observed in our study, with 100% of patients reporting satisfaction and willingness to undergo repeat treatment, exceeds satisfaction and retreatment intent reported in earlier monopolar RF experiences and aligns with outcomes seen with newer‐generation RF technologies. The improved tolerability of the DENSITY treatment (with no significant pain among study participants) is likely the explanation for this difference. Pain has historically been a limiting factor for older non‐invasive skin tightening technologies, often requiring analgesia and leading to suboptimal patient acceptance [3]. Our results indicate that modern RF protocols with optimized energy delivery and cooling can largely mitigate this concern, making the experience comfortable without compromising efficacy.

Subgroup analyses provided additional insights into treatment response variability. Older patients (> 55 years) demonstrated a noticeably higher proportion of marked improvement and ’very satisfied’ responses compared to younger patients, despite similar treatment parameters. This observation is consistent with previously reported RF outcomes in mature skin, where greater baseline laxity may allow more visually appreciable tightening. Correlation analyses further showed that perceived visible improvement strongly tracked satisfaction and willingness to recommend, highlighting the importance of subjective outcomes in evaluating aesthetic RF treatment.

The safety profile observed in this study was excellent, consistent with the generally low complication reported for non‐ablative RF treatments. In a large 5700 monopolar RF facial tightening sessions, serious adverse events were rare, and only mild, transient effects were noted in most cases [10]. Similarly, we detected no significant adverse events in 25 treatments, with only transient erythema or swelling in a few patients. The lack of downtime for 92% of our patients highlights a key advantage of this procedure over more invasive modalities. The sequential dual‐frequency design with real‐time impedance feedback may further enhance safety by delivering controlled thermal doses to each layer without overheating the epidermis. Although our follow‐up was short‐term, earlier research indicates that RF‐induced dermal remodeling can continue for several months post‐treatment [3]. A larger follow‐up might reveal even greater improvement or sustained benefits beyond the 3‐month point observed here.

This study has limitations inherent to its retrospective design. There was no control group or randomization, and the sample size was modest. The data were pooled from multiple centers, introducing potential variability in technique despite a standardized protocol. Outcomes were assessed by unblinded treating providers and by the patients themselves, which could introduce subjective bias. Additionally, the study relied on qualitative improvement scales and patient surveys; more objective measures of skin tightening were not utilized. These factors may affect the precision and generalizability of the findings. Although exploratory subgroup and correlation analyses were performed, the limited sample size in several subcategories (particularly younger ages) constrains inferential robustness.

## 5 Conclusion

The results of this multicentre retrospective study indicate that the DENSITY NOIR sequential monopolar‐bipolar RF treatment is an effective and well‐tolerated method for nonsurgical dermatologic aesthetic improvement. Significant improvements in skin firmness and texture were achieved in the majority of patients after a single treatment, with high patient satisfaction and minimal pain or downtime. These findings highlight the benefit of delivering RF energy to multiple skin depths in a single session, thereby addressing both the deep and superficial components of skin aging. While further research (including prospective controlled trials and long‐term evaluations) is warranted, our real‐world data support the use of the dual‐mode DENSITY RF device as a valuable addition to the armamentarium for skin rejuvenation, offering patients appreciable results with minimal disruption to daily life.

## Author Contributions

All authors equally contributed to the study and the manuscript development.

## Funding

The authors have nothing to report.

## Ethics Statement

The study was conducted in accordance with the Principles of the Declaration of Helsinki and Good Clinical Practices, and all subjects provided written informed consent prior to inclusion.

## Consent

The authors confirm that all required patient consent for the use of photographs was obtained prior to publication.

## Conflicts of Interest

Narendra Kumar is a consultant to Jeisys Med Inc., Seoul, South Korea. The authors declare no conflicts of interest in this work.

## Data Availability

The data that support the findings of this study are available from the corresponding author upon reasonable request.
